# Physicians' Drug Information-Seeking Behavior at King Abdulaziz Medical City, Western Region: A Cross-Sectional Study

**DOI:** 10.7759/cureus.109619

**Published:** 2026-05-25

**Authors:** Ohoud I Qadi, Mohammed Aseeri, Youssif Eman, Khadijah Angawi

**Affiliations:** 1 Department of Pharmaceutical Care Services, King Abdulaziz Medical City, Jeddah, SAU; 2 Department of Clinical Research, King Abdullah International Medical Research Center, Jeddah, SAU; 3 Department of Health Services and Hospital Administration, King Abdulaziz University, Jeddah, SAU; 4 Department of Medicine, King Saud Bin Abdulaziz University for Health Sciences, Jeddah, SAU; 5 Department of Clinical Pharmacy and Pharmaceutical Care, King Abdulaziz Medical City, Jeddah, SAU

**Keywords:** drug information, information-seeking behavior, medication safety, physicians, saudi arabia

## Abstract

Background/objectives: This study aimed to evaluate drug information-seeking behaviors, preferred sources, perceived reliability, and related challenges among physicians at King Abdulaziz Medical City, Western Region (KAMC-WR), Saudi Arabia.

Methods: In this cross-sectional study, we surveyed physicians at KAMC-WR, a tertiary care center in Saudi Arabia’s Western Region, from October 15 to December 15, 2025. Data were collected via a validated, self-administered online questionnaire adapted from prior studies. The survey evaluated demographics, drug information needs, seeking behaviors, challenges, and the reliability of sources. Physicians were categorized as passive or active information seekers based on the number of clinical situations in which they sought drug-related information, with passive seekers reporting searches in zero to one clinical situation and active seekers reporting searches in two or more clinical situations. Data were analyzed using descriptive statistics, chi-square/Fisher’s exact tests, the Mann-Whitney U test, and logistic regression in SPSS version 28. A p-value of <0.05 was considered statistically significant.

Results: Among 148 responding physicians, most searched for drug information daily or weekly. Frequently sought details included dosage regimens (146, 98.6%), adverse effects (138, 93.2%), and contraindications (138, 93.2%). Websites and clinical pharmacists were the most commonly used sources, while mobile apps were preferred for accessibility. Key challenges included polypharmacy management, source variability, and restricted subscription access. Specialists and consultants demonstrated higher odds of being active seekers compared with residents (adjusted OR=12.5, 95% CI: 1.66-93.89, p=0.014).

Conclusions: In this study conducted at KAMC-WR, physicians frequently sought drug information during routine clinical practice, particularly through digital resources, but encountered access and workflow barriers. Improving institutional drug information resources and pharmacist collaboration could promote safer and more effective prescribing.

## Introduction

Most diseases and health conditions are primarily managed with pharmacotherapy, placing physicians at the center of prescribing decisions [1,2]. Rational medication use requires selecting the appropriate medication, dose, route, and duration for the correct patient to achieve optimal therapeutic outcomes while minimizing harm [1]. Achieving these outcomes depends on physicians’ clinical expertise and their ability to access and apply evidence-based drug information [3].

The growing complexity of pharmacotherapy and the large volume of available drug information make it difficult for physicians to consistently rely on up-to-date and reliable sources [4]. In busy clinical settings, this may lead to the use of more convenient but less reliable sources, which can affect prescribing decisions and patient safety [1,4].

In response, digital health technologies, particularly the emergence of artificial intelligence-driven tools, are increasingly transforming how healthcare professionals access and utilize information [5,6]. In Saudi Arabia, this issue is especially important due to recent changes in the healthcare system, including the expansion of digital health services and ongoing reforms under Vision 2030. These changes may influence how physicians search for and use drug information, making it important to understand current practices in this setting.

Previous studies have shown that physicians often use a mix of online resources, colleagues, and clinical guidelines when searching for drug information [1,7,8]. However, these patterns can vary depending on experience, specialty, and access to resources. Although some studies from Saudi Arabia have examined physicians’ drug information-seeking behavior and awareness of drug information services, the available evidence remains limited, particularly in tertiary care settings and in the context of ongoing healthcare changes and the increasing use of digital health resources [2]. Therefore, this study aims to provide insight into how physicians in a tertiary care setting in Saudi Arabia access and use drug-related information in their daily practice.

## Materials and methods

Study design and sample

An observational, cross-sectional study was conducted at King Abdulaziz Medical City, Western Region (KAMC-WR) in Jeddah, Saudi Arabia. Following approval from the King Abdullah International Medical Research Center (KAIMRC) Biomedical Ethics Research Committee (reference number: NRJ25/059/5), data were collected over two months, from October 15, 2025, to December 15, 2025, using an online, self-administered questionnaire developed using Microsoft Forms.

The study aimed to analyze drug information-seeking behavior among physicians working at KAMC-WR, Saudi Arabia. The study population comprised physicians employed at KAMC-WR, a large tertiary care institution with multiple specialized centers and an approximate capacity of 750 beds, located in Jeddah, Saudi Arabia. Participants were selected using census sampling, and the survey link was distributed via institutional email to all physicians working at KAMC-WR during the study period, irrespective of specialty or professional role. This approach was selected to maximize participation and ensure broad representation across physician groups within the institution. Reminder emails were sent biweekly to improve the response rate. In addition, a statement at the beginning of the survey informed participants that completing the questionnaire would qualify them for entry into a gift card draw, serving as a noncoercive incentive.

According to records from the Western Region Office of Medical Services, 675 physicians were employed at KAMC-WR during the study period. Assuming a 95% confidence level, a ±5% margin of error, and a conservative response distribution of 50%, the minimum required sample size was estimated to be 246 participants using the Raosoft sample size calculator [9]. Because the survey targeted all eligible physicians within a single institution using census sampling, no design effect was applied.

Measurement tool and data analysis

Data were collected using a validated questionnaire adapted from Al Zoubi et al. [1]. The instrument was translated from Arabic into English by a certified translation office. The original questionnaire had previously undergone expert face and content validation as well as pilot testing. In the current study, the internal consistency reliability of the Likert-scale domains was assessed using Cronbach’s alpha. The challenges domain demonstrated acceptable internal consistency (10 items; α=0.710), while the reliability of drug information sources domain demonstrated good internal consistency (11 items; α=0.788). Although no formal construct revalidation was performed following translation and adaptation, the original questionnaire structure was maintained, and a standardized electronic format was used to provide all participants with identical instructions and minimize response bias.

The questionnaire comprised multiple-choice, checkbox, and Likert-scale items and was structured into six sections assessing the following aspects: physicians’ demographic and professional characteristics; drug information needs; situations requiring drug information; information-seeking behaviors; challenges encountered during information retrieval; and the perceived reliability of drug information sources (Appendix A).

The first section collected demographic and professional data, including sex, age, years of professional experience, country in which the bachelor’s degree was obtained, language of instruction during undergraduate training, history of academic employment, job title, clinical specialty, country awarding the most recent specialty qualification, and frequency of drug information seeking. The second section assessed physicians’ drug information needs, including the types of information required and the availability of internet access in the workplace. The third section examined clinical situations in which drug information was sought. The fourth section evaluated physicians’ information-seeking behaviors, including preferred sources, formats, and access methods. The final section explored barriers to obtaining drug information and physicians’ perceptions of the reliability of available drug information sources.

The first page of the online questionnaire presented a concise summary of the study objectives. Participants were informed that participation was voluntary and that they could withdraw at any time without providing a reason. They were assured that all responses would remain anonymous and confidential. Electronic informed consent was obtained from all participants before survey initiation.

Dependent variables

Physicians’ drug information-seeking behavior served as the dependent variable. This construct was assessed using participants’ responses regarding the clinical contexts in which they sought drug-related information, including patient counseling, medication history review, prescribing decisions, ward rounds, and updating clinical knowledge. Physicians were classified as passive or active information seekers based on the number of clinical situations in which they reported searching for drug-related information. Those who reported 0-1 situations were considered passive seekers, while those who reported ≥2 situations were considered active seekers. This approach was based on previous literature that distinguishes between limited and more frequent engagement in information-seeking behavior [1,5,10]. As there is no standard definition for this classification, it was used to provide a practical method for comparing different levels of engagement.

Independent variables

Physicians’ demographic and professional characteristics, perceived challenges encountered during drug information searching, and perceived reliability of information sources were treated as independent variables. Demographic and professional characteristics included age group, sex, years of professional experience, job title, and prior employment in the academic sector. Challenges related to drug information searching were assessed using multiple items addressing workload, time constraints, reliability and availability of information sources, access to subscription-based resources, language barriers, technological difficulties, and information overload. Each item was rated on a Likert scale (1=disagree, 2=neutral, 3=agree), and responses were summed to generate a total challenge score, with higher scores indicating greater perceived barriers. The perceived reliability of drug information sources, including colleagues, pharmacists, clinical pharmacy specialists, clinical guidelines, specialized websites, and institutional intranet resources, was also measured using a Likert scale (1=not at all, 2=a little, 3=average, 4=very much, 5=mostly). Item scores were aggregated to produce a total reliability score, with higher scores reflecting greater perceived reliability. For survey questions allowing multiple responses (e.g., sources of drug information and clinical situations requiring information), each response option was treated as an independent binary variable (selected=1, not selected=0) and analyzed descriptively as frequencies and percentages.

Analysis methods

Statistical analyses were conducted using SPSS version 28 (IBM Corp., Armonk, NY, USA). The Kolmogorov-Smirnov test was applied to assess the normality of numerical variables. As the data were non-normally distributed, numerical variables were summarized using medians and interquartile ranges (Q1, Q3) and analyzed using the Mann-Whitney U test. Categorical variables were expressed as frequencies and percentages and compared using the chi-square or Fisher’s exact test, as appropriate. Logistic regression analysis was performed to identify factors associated with drug information-seeking status. Statistical significance was defined as a two-tailed p-value<0.05.

## Results

A total of 675 physicians were invited to participate in the study; 148 completed the survey, yielding a response rate of 21.9%. The sample comprised 148 physicians (99 male and 49 female), of whom 81 (54.7%) were aged ≤30 years. Most participants, 117 (79.1%), had <10 years of professional experience, and 121 (81.8%) had obtained their bachelor’s degree in Saudi Arabia. English was reported as the primary language of instruction by 97 (65.5%) respondents. Nearly half of the participants, 68 (45.9%), were employed in academic settings. Residents constituted the majority of respondents, 81 (54.7%), followed by specialists or consultants, 50 (33.8%). Family medicine was the most common specialty, representing 60 (40.5%) of the sample, and 115 (77.7%) physicians had obtained their most recent specialty qualification in Saudi Arabia. Regarding information-seeking frequency, 72 (48.6%) physicians reported searching for drug information daily, whereas 61 (41.2%) did so weekly (Table 1).

**Table 1:** Baseline characteristics of the studied participants Data are presented as frequency (n) and percentage (%). ICU, intensive care unit

Item	Total participants (n=148)
Age (years)	
<30	81 (54.7%)
30-39	38 (25.7%)
40-49	17 (11.5%)
50-59	9 (6.1%)
≥60	3 (2%)
Sex	
Male	99 (66.9%)
Female	49 (33.1%)
Experience (years)	
<10	117 (79.1%)
10-19	14 (9.5%)
20-29	12 (8.1%)
≥30	5 (3.4%)
Country in which the Bachelor’s degree was obtained	
Saudi Arabia	121 (81.8%)
Other Arab countries	4 (2.7%)
Europe	1 (0.7%)
UK	1 (0.7%)
USA	2 (1.4%)
Other	19 (12.8%)
Language of instruction in the country of study	
English	97 (65.5%)
Arabic	49 (33.1%)
Other	2 (1.4%)
Previously or currently worked in the academic sector	
No	80 (54.1%)
Yes	68 (45.9%)
Job title	
Intern	9 (6.1%)
Resident	81 (54.7%)
General physician	8 (5.4%)
Specialist/consultant	50 (33.8%)
Specialization	
Internal medicine	20 (13.5%)
Pediatrics	8 (5.4%)
General surgery	5 (3.4%)
Anesthesiology	5 (3.4%)
ICU	4 (2.7%)
Family medicine	60 (40.5%)
Emergency medicine	14 (9.5%)
Intern	3 (2%)
Other	29 (19.6%)
The country granting the most recent specialty degree	
Saudi Arabia	115 (77.7%)
Other Arab countries	1 (0.7%)
Europe	4 (2.7%)
UK	7 (4.7%)
USA	3 (2%)
Canada	7 (4.7%)
Other	11 (7.4%)
Frequency of drug information seeking	
Daily	72 (48.6%)
Weekly	61 (41.2%)
Monthly	11 (7.4%)
Annually	3 (2%)
Less than that	1 (0.7%)

Drug information needed by physicians

**Figure 1:**
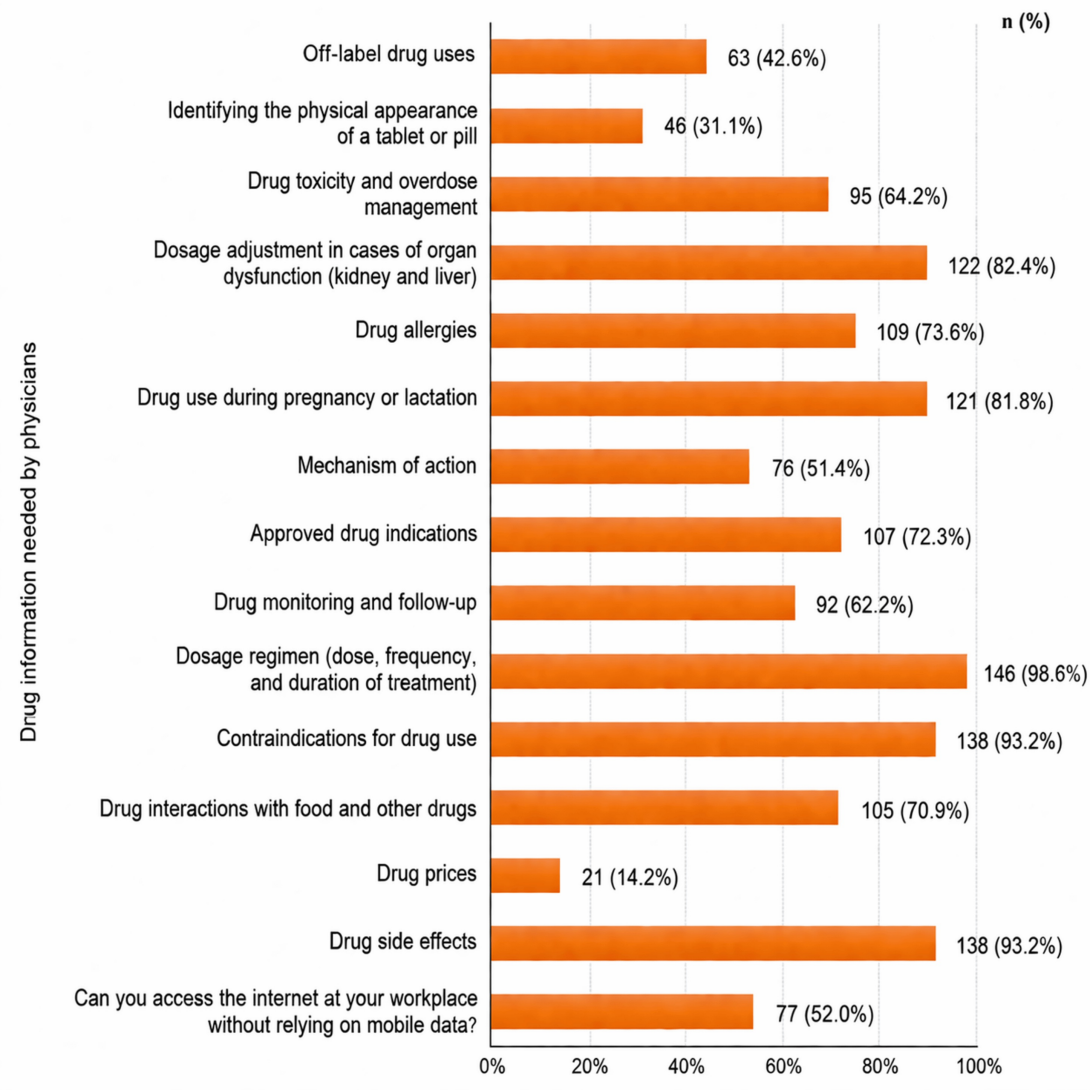
Drug information needed by physicians Data are presented as frequency (n) and percentage (%).

The types of drug information sought by physicians are presented in Figure 1. The most frequently searched category was dosage regimen, including dose, frequency, and treatment duration, reported by 146 (98.6%) of respondents. Information on drug adverse effects and contraindications was sought by 138 (93.2%) of physicians. Dosage adjustment in organ dysfunction, particularly renal and hepatic impairment, was reported by 122 (82.4%), whereas drug use during pregnancy or lactation was reported by 121 (81.8%). Additional commonly sought information included drug allergies, reported by 109 (73.6%); approved indications, reported by 107 (72.3%); and interactions with food or other medications, reported by 105 (70.9%).

Physicians’ behaviors in searching for information

Drug information was most commonly required during prescribing, reported by 125 (84.5%) of physicians, followed by patient counseling, 92 (62.2%), medication history review, 90 (60.8%), and updating clinical knowledge, 87 (58.8%). The most frequently used sources for obtaining drug-related information were websites 112 (75.7%) and pharmacists or clinical pharmacy specialists 74 (50%). Mobile applications 119 (80.4%) and websites 96 (64.9%) were the preferred formats for accessing drug information. When using the Ministry of National Guard-Health Affairs (MNGHA) intranet, 91 (61.5%) of physicians relied on institutional guidelines and protocols. Intranet resources were accessed daily by 35 (23.6%) respondents and several times per week by 30 (20.3%) respondents. The perceived usefulness of intranet resources was rated as slight by 39 (26.4%), moderate by 43 (29.1%), and high by 39 (26.4%) physicians. Websites were used by the majority, 112 (75.7%), to obtain drug information, and the most commonly used website was UpToDate, 129 (87.2%). Workplace access to subscription-based websites and journals was available to 77 (52%) physicians. Mobile applications were used by 117 (79.1%) respondents to perform clinical calculations, such as drug dosing, renal function estimation, and body mass index calculation. Additionally, 59 (39.9%) physicians stated that they provide patients with all the necessary information when asked to recommend reliable medication-related information sources (Table 2).

**Table 2:** Physicians’ behaviors in searching for information Data are presented as frequency (n) and percentage (%). Multiple responses were allowed; percentages do not sum to 100%. BMI, body mass index; MNGHA, Ministry of National Guard Health Affairs; DPP, departmental policy and procedure; APP, administrative policies and procedures; IV, intravenous; SANG-HA, Saudi National Guard Health Affairs; SFDA, Saudi Food and Drug Authority; PDF, portable document format;

Item	Total participants (n=148)
Situations when drug information is needed	
During patient counseling	92 (62.2%)
When reviewing a patient’s medication history	90 (60.8%)
While prescribing medication	125 (84.5%)
During ward rounds	60 (40.5%)
When updating clinical knowledge	87 (58.8%)
During interdisciplinary discussions	37 (25%)
Other	2 (1.4%)
Preferred sources to obtain drug-related information	
Colleagues	56 (37.8%)
Websites	112 (75.7%)
Research articles	54 (36.5%)
Pharmacists and clinical pharmacy specialists	74 (50%)
Medical books	51 (34.5%)
MNGHA intranet	47 (31.8%)
National drug formulary	27 (18.2%)
Drug leaflet	21 (14.2%)
Pharmacopoeias (USA, UK)	12 (8.1%)
Global clinical practice guidelines and recommendations	45 (30.4%)
Conferences, seminars, and courses	12 (8.1%)
Other	5 (3.4%)
Preferred format for accessing drug information	
Mobile applications	119 (80.4%)
Websites	96 (64.9%)
PDF or downloadable documents	29 (19.6%)
Consultation with pharmacists or colleagues	38 (25.7%)
Integrated hospital intranet resources	44 (29.7%)
Printed handbooks/manuals	21 (14.2%)
Other	1 (0.7%)
MNGHA intranet resources used	
Guidelines and protocol	91 (61.5%)
Allergy algorithm	12 (8.1%)
Drug safety alert	35 (23.6%)
DPP and APP	22 (14.9%)
Antidote	11 (7.4%)
Medication restriction list	17 (11.5%)
Non-formulary	9 (6.1%)
IV manual	21 (14.2%)
SANG-HA formulary	23 (15.5%)
Medical calculator	22 (14.9%)
Adverse drug reaction	25 (16.9%)
SFDA risk minimization measures	14 (9.5%)
Compounding list	2 (1.4%)
Frequency of using intranet resources	
Daily	35 (23.6%)
Several times a week	30 (20.3%)
Weekly	19 (12.8%)
Monthly	18 (12.2%)
Rarely	31 (20.9%)
Never	15 (10.1%)
Overall usefulness rating of MNGHA intranet resources	
Not useful at all	15 (10.1%)
Slightly useful	39 (26.4%)
Moderately useful	43 (29.1%)
Very useful	39 (26.4%)
Extremely useful	12 (8.1%)
Websites used to search for drug information	
Lexicomp	24 (16.2%)
UpToDate	129 (87.2%)
Medscape	65 (43.9%)
The first website that appears on Google	17 (11.5%)
MNGHA.com	16 (10.8%)
DailyMed	9 (6.1%)
Clinicaltrials.gov	8 (5.4%)
AMBOSS	5 (3.4%)
Drugs.com	23 (15.5%)
Micromedex	13 (8.8%)
FDA or SFDA	25 (16.9%)
Other	11 (7.4%)
How do you access paid-subscription journals/websites?	
My workplace provides access to most websites and journals	77 (52%)
I use Sci-Hub	30 (20.3%)
I pay for a personal subscription	42 (28.4%)
I ask a colleague with a subscription for help	18 (12.2%)
I searched for the same information in free sources	24 (16.2%)
I contacted our institutional librarian	5 (3.4%)
My workplace provides access to a limited number of websites and journals	41 (27.7%)
I use a colleague’s account subscribed to these journals/websites	7 (4.7%)
I do not need information from journals or websites that require a paid subscription	4 (2.7%)
Other	1 (0.7%)
How do you perform calculations (drug dosage, kidney function, BMI, etc.)?	
Using a calculator	68 (45.9%)
Using mobile applications	117 (79.1%)
Consulting a pharmacist/clinical pharmacist	43 (29.1%)
Asking a colleague for help	24 (16.2%)
I use specialized websites for this purpose	27 (18.2%)
I do not need to perform any calculations in my specialty	3 (2%)
Other (Using best care calculator)	1 (0.7%)
If a patient asks you for a reliable source to learn more about their medication	
It depends on the patient’s educational background	39 (26.4%)
I do not recommend any source to avoid errors that may result from patients searching for information on their own without consulting or referring to a doctor to verify its accuracy	14 (9.5%)
I ask them to read the drug leaflet from the SFDA website	8 (5.4%)
I refer them to a pharmacist/clinical pharmacist	23 (15.5%)
I provide my patients with all the necessary information	59 (39.9%)
I ask them to search for the information they need using Google	4 (2.7%)
Other	1 (0.7%)

Challenges faced by physicians when searching for drug information

Most participants reported that the primary challenges encountered when searching for drug information included managing a large number of medications, reported by 88 (59.5%); excessive variability among available information sources, reported by 63 (42.6%); and limited access to journals and websites requiring costly subscriptions, reported by 61 (41.2%). The median (Q1, Q3) total challenge score was 20.5 (18, 23) (Table 3).

**Table 3:** Challenges faced by physicians when searching for drug information Data are presented as frequency (n) and percentage (%). Total score is presented as median (Q1, Q3).

Item	Disagree	Neutral	Agree
I handle a large number of medications	9 (6.1%)	51 (34.5%)	88 (59.5%)
There is a lack of drug information	36 (24.3%)	79 (53.4%)	33 (22.3%)
There is a lack of reliable references and sources for obtaining drug information	55 (37.2%)	60 (40.5%)	33 (22.3%)
Language barrier	97 (65.5%)	36 (24.3%)	15 (10.1%)
Difficulty using technology	89 (60.1%)	41 (27.7%)	18 (12.2%)
Lack of sufficient time to search for information	37 (25%)	54 (36.5%)	57 (38.5%)
The overwhelming variation in available information sources	26 (17.6%)	59 (39.9%)	63 (42.6%)
Difficulty identifying the most reliable source to use	38 (25.7%)	56 (37.8%)	54 (36.5%)
Limited access to journals and websites requiring a high-cost subscription	22 (14.9%)	65 (43.9%)	61 (41.2%)
Difficulty analyzing and understanding research findings and statistics	34 (23%)	68 (45.9%)	46 (31.1%)
Total score	20.5 (18, 23)		

Reliability of drug information sources from physicians’ perspectives

The highest perceived reliability was reported for information obtained from fellow physicians, pharmacists and clinical pharmacy specialists, specialized websites, clinical practice guidelines, and the MNGHA intranet. The corresponding median (Q1, Q3) scores were 4 (3, 4), 4 (4, 5), 4 (3, 5), 4 (4, 5), and 3.5 (3, 4), respectively, yielding a total reliability score of 38 (35, 42) (Table 4).

**Table 4:** Reliability of drug information sources from doctors’ perspectives Data are presented as frequency (n) and percentage (%). Individual item scores and total score are presented as median (Q1, Q3). MNGHA, Ministry of National Guard-Health Affairs

Item	Not at all	A little	Average	Very much	Mostly	Total score
MNGHA intranet	16 (10.8%)	15 (10.1%)	43 (29.1%)	40 (27%)	34 (23%)	3.5 (3, 4)
Medical sales representatives	40 (27%)	43 (29.1%)	50 (33.8%)	11 (7.4%)	4 (2.7%)	2 (1,3)
Conferences, seminars, and courses	11 (7.4%)	21 (14.2%)	55 (37.2%)	47 (31.8%)	14 (9.5%)	3 (3,4)
Fellow physicians	3 (2%)	6 (4.1%)	52 (35.1%)	59 (39.9%)	28 (18.9%)	4 (3, 4)
Pharmacists and clinical pharmacy specialists	4 (2.7%)	4 (2.7%)	25 (16.9%)	66 (44.6%)	49 (33.1%)	4 (4, 5)
Specialized websites	2 (1.4%)	1 (0.7%)	37 (25%)	56 (37.8%)	52 (35.1%)	4 (3, 5)
Research papers	8 (5.4%)	13 (8.8%)	57 (38.5%)	47 (31.8%)	23 (15.5%)	3 (3, 4)
Medical books	13 (8.8%)	14 (9.5%)	53 (35.8%)	40 (27%)	28 (18.9%)	3 (3, 4)
The national formulary	14 (9.5%)	13 (8.8%)	55 (37.2%)	48 (32.4%)	18 (12.2%)	3 (3, 4)
Clinical guidelines	3 (2%)	5 (3.4%)	27 (18.2%)	54 (36.5%)	59 (39.9%)	4 (4, 5)
Pharmacopoeias (USA, UK)	25 (16.9%)	10 (6.8%)	65 (43.9%)	37 (25%)	11 (7.4%)	3 (3, 4)
Total score	38 (35, 42)					

The relation between drug information seeking and baseline characteristics

Regarding drug information-seeking behavior, physicians were categorized into two groups based on the number of clinical situations in which drug information was sought: passive seekers (0-1 situation) and active seekers (≥2 situations). Participants were classified according to their responses to the question, “In which of the following situations do you typically search for drug information?”

There was no significant relationship between drug information-seeking status and age, sex, years of experience, employment in the academic sector, or job title (Table 5).

**Table 5:** Relation between drug information seeking and baseline characteristics Data are presented as frequency (n) and percentage (%). Statistical tests: Chi-square test or Fisher’s exact test (for cells with an expected count <5) for all categorical variables. A p-value <0.05 was considered statistically significant.

Item	Passive seeker (n=12)	Active seeker (n=136)	Test statistic	P-value
Age (years)				
Under 30	5 (41.7%)	76 (55.9%)	χ²(3)=2.49	0.526
30-39	3 (25%)	35 (25.7%)		
40-49	3 (25%)	14 (10.3%)		
50 or more	1 (8.3%)	11 (8.1%)		
Sex				
Male	9 (75%)	90 (66.2%)	Fisher's exact	0.751
Female	3 (25%)	46 (33.8%)		
Experience (years)				
Under 10	8 (66.7%)	109 (80.1%)	χ²(3)=7.10	0.081
10-19	1 (8.3%)	13 (9.6%)		
20-29	1 (8.3%)	11 (8.1%)		
30 or more	2 (16.7%)	3 (2.2%)		
Previously or currently working in the academic sector				
No	5 (41.7%)	75 (55.1%)	χ²(1)=0.81	0.369
Yes	7 (58.3%)	61 (44.9%)		
Job title				
Intern	2 (16.7%)	7 (5.1%)	χ²(3)=8.40	0.053
Resident	2 (16.7%)	79 (58.1%)		
General physician	1 (8.3%)	7 (5.1%)		
Specialist/consultant	7 (58.3%)	43 (31.6%)		

The relation between drug information seeking and the challenges faced by physicians when searching for drug information

The proportion of active seekers who perceived substantial variation among available information sources was significantly higher than that of passive seekers (P=0.039) (Table 6).

**Table 6:** Relation between drug information seeking and challenges faced by physicians when searching for drug information ^ *^Statistically significant. Categorical variables are presented as frequency (n) and percentage (%). Total score is presented as median (Q1, Q3). Chi-square test or Fisher’s exact test was used for categorical variable comparisons, while the Mann-Whitney U test was used only for total score comparison. P-value <0.05 was considered statistically significant.

Item	Passive seeker (n=12)	Active seeker (n=136)	Test statistic (χ²/U)	P-value
I handle a large number of medications				
Disagree	0 (0%)	9 (6.6%)	χ²(2)=0.99	0.631
Neutral	5 (41.7%)	46 (33.8%)		
Agree	7 (58.3%)	81 (59.6%)		
There is a lack of drug information				
Disagree	2 (16.7%)	34 (25%)	χ²(2)=1.06	0.569
Neutral	6 (50%)	73 (53.7%)		
Agree	4 (33.3%)	29 (21.3%)		
There is a lack of reliable references and sources				
Disagree	3 (25%)	52 (38.2%)	χ²(2)=0.85	0.736
Neutral	6 (50%)	54 (39.7%)		
Agree	3 (25%)	30 (22.1%)		
Language barrier				
Disagree	9 (75%)	88 (64.7%)	χ²(2)=2.10	0.375
Neutral	1 (8.3%)	35 (25.7%)		
Agree	2 (16.7%)	13 (9.6%)		
Difficulty using technology				
Disagree	8 (66.7%)	81 (59.6%)	χ²(2)=0.28	0.923
Neutral	3 (25%)	38 (27.9%)		
Agree	1 (8.3%)	17 (12.5%)		
Lack of sufficient time to search for information				
Disagree	3 (25%)	34 (25%)	χ²(2)=3.33	0.168
Neutral	7 (58.3%)	47 (34.6%)		
Agree	2 (16.7%)	55 (40.4%)		
The overwhelming variation in available information sources				
Disagree	3 (25%)	23 (16.9%)	χ²(2)=6.36	0.039^*^
Neutral	8 (66.7%)	51 (37.5%)		
Agree	1 (8.3%)	62 (45.6%)		
Difficulty identifying the most reliable source to use				
Disagree	3 (25%)	35 (25.7%)	χ²(2)=2.86	0.243
Neutral	7 (58.3%)	49 (36%)		
Agree	2 (16.7%)	52 (38.2%)		
Limited access to journals and websites requiring a high-cost subscription				
Disagree	0 (0%)	22 (16.2%)	χ²(2)=2.56	0.305
Neutral	7 (58.3%)	58 (42.6%)		
Agree	5 (41.7%)	56 (41.2%)		
Difficulty analyzing and understanding research findings and statistics				
Disagree	4 (33.3%)	30 (22.1%)	χ²(2)=1.53	0.502
Neutral	6 (50%)	62 (45.6%)		
Agree	2 (16.7%)	44 (32.4%)		
Total score	19 (17.25, 20.75)	21 (18, 23)	U=964.0	0.297

The relation between drug information seeking and the reliability of drug information sources from physicians' perspectives

There was no significant relationship between drug information seeking and the reliability of drug information sources from doctors’ perspectives (Table 7).

**Table 7:** Relation between drug information seeking and reliability of drug information sources from doctors’ perspectives Data are presented as median (Q1, Q3). Statistical test: Mann-Whitney U test. P-value <0.05 is considered statistically significant. MNGHA, Ministry of National Guard-Health Affairs

Item	Passive seeker (n=12)	Active seeker (n=136)	Test statistic (Mann-Whitney U)	P-value
MNGHA intranet	2.5 (2, 4)	4 (3, 4)	556	0.060
Medical sales representatives	2 (1, 4)	2 (1, 3)	804	0.930
Conferences, seminars, and courses	3 (2.25, 3.75)	3 (3, 4)	726	0.508
Fellow physicians	4 (3, 4)	4 (3, 4)	790	0.846
Pharmacists and clinical pharmacy specialists	4 (3, 4.75)	4 (4, 5)	696	0.366
Specialized websites	4 (3, 4.75)	4 (3, 5)	653	0.224
Research articles	3 (2.25, 4)	3 (3, 4)	650	0.219
Medical books	3 (2.25, 4)	3 (3, 4)	723	0.495
The National Formulary	3 (2.25, 4)	3 (3, 4)	777	0.771
Clinical guidelines	4 (3, 4.75)	4 (4, 5)	660	0.242
Pharmacopoeias (US, British)	3.5 (3, 4)	3 (3, 4)	633	0.173
Total score	38 (28.25, 39.75)	38.5 (35, 42)	693	0.407

Logistic regression analysis for factors associated with active drug information seeking

In the univariate and multivariable logistic regression models, specialists/consultants showed significantly higher odds of seeking drug information than residents did, with an odds ratio (OR) (95% CI) of 8.46 (1.3-55.32, P=0.026) and 12.5 (1.66-93.89, P=0.014), respectively (Table 8).

**Table 8:** Logistic regression analysis for factors associated with active drug information seeking Statistical test: Binary logistic regression (univariate and multivariable). P-value <0.05 is considered statistically significant. OR, odds ratio; Ref, reference category

Item	Univariate analysis			Multivariable analysis		
	Unadjusted OR	95% CI	P-value	Adjusted OR	95% CI	P-value
Age (years)						
<30 (Ref)	1	-	-	1	-	-
≥30	0.56	0.17-1.87	0.348	3.54	0.51-24.74	0.203
Sex						
Male (Ref)	1	-	-	1	-	-
Female	1.53	0.4-5.94	0.536	1.36	0.3-6.23	0.69
Experience (years)						
<10 (Ref)	1	-	-	1	-	-
10-19	0.95	0.11-8.25	0.966	1.76	0.17-18.42	0.635
≥20	0.34	0.08-1.44	0.144	0.56	0.09-3.33	0.524
Previously or currently working in the academic sector						
No (Ref)	1	-	-	1	-	-
Yes	0.58	0.18-1.92	0.374	0.96	0.24-3.83	0.953
Job title						
Resident (Ref)	1	-	-	1	-	-
Specialist/consultant	8.46	1.3-55.32	0.026	12.5	1.66-93.89	0.014
Other	1.32	0.3-5.79	0.716	0.83	0.13-5.13	0.84
Challenge score	1.05	0.9-1.23	0.526	1.03	0.87-1.22	0.719
Reliability score	1.04	0.96-1.13	0.311	1.05	0.96-1.14	0.29

## Discussion

This study investigated the drug information-seeking behavior of physicians, regardless of their seniority or specialty, at KAMC-WR. It demonstrates that most physicians search for drug information in routine clinical practice, especially while prescribing, on a daily or weekly basis. This finding is consistent with prior research identifying drug information as one of the most frequently sought categories of clinical knowledge [2,7,11]. Other studies suggest that evidence-based drug information is essential for rational prescribing, clinical decision-making, and reducing medication errors and adverse drug events, highlighting the importance of the availability of drug information sources during clinical practice [4,5,7,12-14].

The highest demand was for information on dosage regimens, adverse drug reactions, contraindications, and medication use in special populations. Several studies showed similar findings, reflecting the growing complexity of pharmacotherapy and the expanding range of available medications [1,11]. Despite the importance of medication pricing and its economic impact on patients and the healthcare system, only 21 (14.2%) participants searched for this type of information. This may be a result of KAMC-WR being a governmental hospital where medications are provided free of charge to eligible patients. Similar findings in previous studies, in which few physicians searched for pricing information, highlight the global need to improve awareness of drug costs [1].

Web-based resources were the most frequently used sources of drug information, followed by consultations with pharmacists and clinical pharmacy specialists. This pattern aligns with findings from studies conducted in Saudi Arabia and other international settings, which indicate that physicians increasingly rely on online databases because of their accessibility, rapid retrieval, and provision of current evidence [1,11]. Despite the subscription cost of the UpToDate database, it was used by 129 (87.2%) participants. This may be attributed to the free access provided through the MNGHA intranet and its status as a trusted, evidence-based clinical reference among healthcare professionals. Similarly, physicians in other studies were more likely to use UpToDate for information searching because of its ease of use and support for clinical decision-making [1,2].

Although the MNGHA intranet received a relatively high reliability score, its use was low, possibly because of factors such as accessibility, familiarity, and perceived comprehensiveness. Physicians may prefer widely used decision-support tools like UpToDate because of their structured content, frequent updates, and user-friendly design. Enhancing accessibility and integration may improve the utilization of the MNGHA intranet.

Pharmacists and clinical pharmacy specialists were perceived as highly reliable sources of drug information, consistent with previous literature highlighting their essential role in promoting rational prescribing and medication safety [2,8]. However, despite this high level of perceived reliability, pharmacists were not consistently the first source consulted, likely because of a preference for rapid online access, workflow constraints, and time pressures in clinical settings. Similar findings in previous studies suggest that structural and organizational factors may limit interdisciplinary consultation despite recognition of pharmacists’ expertise [4].

Mobile applications emerged as the preferred format for accessing drug information, reflecting physicians’ need for rapid and efficient decision support at the point of care. These findings are consistent with previous studies showing an increasing reliance on digital resources in clinical practice [6,15]. However, variability in source reliability remains a concern, particularly in settings where access to subscription-based databases is limited.

Despite the broad availability of drug information resources, physicians in this study reported multiple challenges when searching for medication-related information. The most frequently identified barriers included managing a large number of medications, variability among available sources, and restricted access to subscription-based journals. These findings are consistent with prior studies that have identified information overload and financial barriers as key impediments to effective drug information seeking [1,4,7].

Notably, perceived information overload was significantly associated with active drug information seeking (p<0.05). This association suggests that physicians who are more engaged in clinical decision-making may be more cognizant of both the volume and complexity of available drug-related information. Similar patterns have been reported previously, indicating that greater exposure to complex clinical scenarios heightens awareness of information-related challenges [4].

Although a descriptive trend suggested that older and more experienced physicians sought drug information less frequently, this was not statistically significant. Similarly, while residents appeared more likely than specialists/consultants to be active information seekers, this difference did not reach significance (p=0.053). However, after adjustment for potential confounders, specialists and consultants were significantly more likely than residents to engage in active drug information-seeking behavior (adjusted OR=12.5, p<0.05), indicating that professional role and clinical responsibility substantially influence this behavior. The wide CIs observed in the regression analysis likely reflect the modest sample size and imbalance between comparison groups.

These findings are consistent with evidence suggesting that senior physicians may seek information more actively because of their involvement in complex cases and accountability for clinical outcomes [5]. In contrast, some studies have reported higher information-seeking frequency among junior physicians, attributed to their limited clinical experience [7,11]. Such discrepancies may reflect differences in study settings, resource availability, or variations in roles and responsibilities between senior and junior physicians.

Future research should include multicenter studies with larger, nationally representative samples to enhance generalizability. Additionally, studies are needed to assess the impact of targeted strategies on prescribing practices and medication safety outcomes.

Implications for practice

These results highlight the need to improve access to reliable drug information sources within healthcare institutions. In addition, strengthening the role of clinical pharmacists and providing training on evaluating information sources may help support safer prescribing practices.

Recommendations

Based on the study findings, several recommendations can be proposed to enhance physicians’ access to and effective utilization of drug information resources. Healthcare institutions should ensure the consistent availability of reliable, evidence-based information resources while simultaneously improving the quality and usability of institutional intranet systems to support efficient access in daily clinical practice. In addition, structured consultation processes should be implemented, with greater integration of pharmacists and clinical pharmacy specialists into multidisciplinary teams to facilitate timely, accurate, and patient-centered drug information support.

Furthermore, targeted educational initiatives are essential to strengthen physicians’ competencies in information retrieval, critical appraisal of drug information sources, and the optimal use of available resources. Such efforts would promote more informed clinical decision-making and improve overall prescribing practices. At the system level, healthcare policymakers should also consider workflow modifications and the integration of clinical decision-support tools to minimize the time burden associated with seeking drug information, thereby enhancing efficiency and supporting safer, high-quality patient care.

Limitations of the study

This study has some limitations. The use of a self-reported questionnaire may introduce response bias. In addition, physicians with a greater interest in drug information resources may have been more likely to participate, potentially introducing volunteer response bias. The lower-than-anticipated response rate may limit the generalizability of the findings and increase the risk of nonresponse bias; however, similar response rates have been reported in web-based physician surveys [16]. Furthermore, the classification of information-seeking behavior may oversimplify a complex process. However, it was used to provide a practical approach for comparing levels of engagement among participants.

## Conclusions

In conclusion, physicians at KAMC-WR frequently seek drug-related information as part of routine clinical practice, particularly during prescribing and patient care activities, with most searches occurring on a daily or weekly basis. The most commonly sought information includes dosage regimens, adverse drug reactions, contraindications, and medication use in special populations, reflecting the complexity of pharmacotherapy and the importance of access to accurate and timely drug information in clinical practice. Digital resources, especially websites and mobile applications, were the most commonly used sources, while pharmacists and clinical pharmacy specialists were perceived as highly reliable.

Several barriers to drug information retrieval were also reported, including challenges related to polypharmacy, variability among available information sources, and limited access to subscription-based resources. Specialists and consultants were more likely to report active drug information-seeking behavior than residents. These findings highlight potential areas for improving access to reliable drug information resources, strengthening the role of pharmacists in clinical practice, and optimizing institutional systems to support efficient information retrieval.

## Appendices

Appendix A 

**Table 9:** The validated questionnaire

Demographic information and participant characteristics																			
1. Sex			☐ Male								☐ Female								
2 . Age	☐ Under 30		☐ 30 - 39		☐ 40 - 49						☐ 50 - 59								☐ 60 or more
3. Years of Experience			☐ Under 10			☐ 10 - 19				☐ 20 - 29						☐ 30 or more			
4. Country of Bachelor's Degree	☐ Saudi Arabia		☐ Other Arab Countries			☐ Europe		☐ UK			☐ USA			☐ Canada					☐ Other
5. Language of Instruction in the Country of Study			☐ English						☐ Arabic						☐ Other				
6. Previously or Currently Worked in the Academic Sector			☐ Yes								☐ No								
7. Job Title			☐ Intern			☐ Resident Physician				☐ General Physician						☐ Specialist / Consultant			
8. Specialization			☐ Internal Medicine ☐ Pediatrics ☐ General Surgery			☐ Obstetrics and Gynecology ☐ Psychiatry ☐ Rheumatology				☐ Otolaryngology (ENT) ☐ Orthopedic Surgery ☐ Ophthalmology						☐ Family Medicine ☐ Dermatology and Venereology ☐ Other:			
9. Country Granting Your Most Recent Specialty Degree	☐ Saudi Arabia		☐ Other Arab Countries			☐ Europe		☐ UK			☐ USA			☐ Canada					☐ Other
10. Need to Search for Drug Information	☐ Daily		☐ Weekly		☐ Monthly						☐ Annually								☐ Less than that
Drug Information Needed by Physicians																			
11. Can you access the internet at your workplace without relying on mobile data?			☐ Yes								☐ No								
12. You usually search for the following information:			Yes								No								
Drug side effects			☐								☐								
Drug prices			☐								☐								
Drug interactions with food and other drugs			☐								☐								
Contraindications for drug use			☐								☐								
Dosage regimen (dose, frequency, and duration of treatment)			☐								☐								
Drug monitoring and follow-up			☐								☐								
Approved drug indications			☐								☐								
Mechanism of action			☐								☐								
Drug use during pregnancy or lactation			☐								☐								
Drug allergies			☐								☐								
Dosage adjustment in cases of organ dysfunction (kidney and liver)			☐								☐								
Drug toxicity and overdose management			☐								☐								
Identifying the physical appearance of a tablet or pill			☐								☐								
Off-label drug uses			☐								☐								
Situations When Drug Information is Needed																			
13. In which of the following situations do you typically search for drug information? (You may select more than one option.)																			
☐ During patient counseling ☐ While prescribing medication		☐ When reviewing a patient's medication history ☐ During ward rounds										☐ When updating clinical knowledge ☐ During interdisciplinary discussions ☐ Other (please specify): ___________							
Physicians' Behaviors in Searching for Information																			
14. Which of the following sources do you prefer or choose to obtain various drug-related information? (You may select more than one option.)																			
☐ Websites ☐ Colleagues ☐ Research papers ☐ Medical books ☐ MNGHA Intranet			☐ Pharmacists and clinical pharmacy specialists ☐ Conferences, seminars, and courses ☐ Drug leaflet ☐ Medical sales representatives								☐ National Drug Formulary ☐ Global clinical practice guidelines and recommendations ☐ Pharmacopoeias (U.S., British) ☐ Other (Specify):								
15. What is your preferred format for accessing drug information? (You may select more than one option.)																			
☐ Printed handbooks/manuals ☐ Mobile applications			☐ Websites ☐ Integrated hospital intranet resources								☐ Consultation with pharmacists or colleagues ☐ PDF or downloadable documents ☐ Other (please specify): ___________								
16. If you use MNGHA Intranet, which of the following have you used or are currently using to search for drug information? (You may select more than one option.)																			
☐ IV Manual ☐ Guidelines and Protocol ☐ SANG-HA Formulary ☐ Non-Formulary ☐ Allergy Algorithm					☐ Medication Restriction List ☐ Compounding List ☐ DPP & APP ☐ Drug Safety Alert ☐ Antidote								☐ Saudi-FDA Risk Minimization Measures ☐ Medical Calculator ☐ Adverse Drug Reaction						
Frequency of Using Intranet Resources																			
☐ Daily	☐ Several times a week				☐ Weekly		☐ Monthly						☐ Rarely					☐ Never	
18. How would you rate the overall usefulness of the MNGHA intranet resources for obtaining drug information?																			
☐ Not useful at all	☐ Slightly useful			☐ Moderately useful			☐ Very useful						☐ Extremely useful						
19. If you use websites, which of the following have you used or are currently using to search for drug information? (You may select more than one option.)																			
☐ I do not search websites ☐ The first website that appears on Google ☐ Clinicaltrials.gov	☐ UpToDate ☐ Medscape ☐ Lexicomp		☐ DailyMed ☐ Drugs.com ☐ Altibbi.com		☐ FDA or SFDA ☐ Rxlist.com ☐ MNGHA.com						☐ Micromedex ☐ Jodrugs.com ☐ Other								
20. How do you access information from journals and/or websites that require a paid subscription? (You may select more than one option.)																			
☐ My workplace provides access to most websites and journals. ☐ My workplace provides access to a limited number of websites and journals. ☐ I pay for a personal subscription. ☐ I ask a colleague with a subscription for help. ☐ I use a colleague's account subscribed to these journals/websites.					☐ I use Sci-Hub. ☐ I search for the same information in free sources. ☐ I do not need information from journals or websites that require a paid subscription. ☐ I contact our institutional librarian. ☐ Other:														
21. If you need to perform calculations (e.g., drug dosage, kidney function, BMI, etc.), how do you do it? (You may select more than one option.)																			
☐ Using a calculator. ☐ Using mobile applications. ☐ Asking a colleague for help. ☐ Consulting a pharmacist / clinical pharmacist.					☐ I use specialized websites for this purpose. ☐ I do not need to perform any calculations in my specialty. ☐ Other:														
22. If a patient asks you for a reliable source to learn more about their medication:																			
☐ I provide my patients with all the necessary information. ☐ I do not recommend any source to avoid errors that may result from patients searching for information on their own without consulting or referring to a doctor to verify its accuracy.					☐ It depends on the patient’s educational background. ☐ I refer them to a pharmacist / clinical pharmacist. ☐ I ask them to search for the information they need using Google. ☐ I ask them to read the drug leaflet from the SFDA website. ☐ Other:														
23. Based on your clinical practice in your specialty:	Agree				Neutral						Disagree								
I handle a large number of medications.	☐				☐						☐								
There is a lack of drug information.	☐				☐						☐								
There is a lack of reliable references and sources for obtaining drug information.	☐				☐						☐								
Challenges faced by physicians when searching for drug information																			
24. Below are the challenges physicians face in searching for drug information:	Agree				Neutral						Disagree								
Language barrier.	☐				☐						☐								
Difficulty using technology.	☐				☐						☐								
Lack of sufficient time to search for information.	☐				☐						☐								
The overwhelming variation in available information sources.	☐				☐						☐								
Difficulty identifying the most reliable source to use.	☐				☐						☐								
Limited access to journals and websites requiring a high-cost subscription.	☐				☐						☐								
Difficulty analyzing and understanding research findings and statistics.	☐				☐						☐								
The reliability of drug information sources from doctors' perspectives.																			
25. In your opinion, how reliable are the following sources for drug information?	Mostly		Very much		Average						A little						Not at all		
MNGHA intranet	☐		☐		☐						☐						☐		
Medical sales representatives	☐		☐		☐						☐						☐		
Conferences, seminars, and courses	☐		☐		☐						☐						☐		
Fellow physicians	☐		☐		☐						☐						☐		
Pharmacists and clinical pharmacy specialists	☐		☐		☐						☐						☐		
Specialized websites	☐		☐		☐						☐						☐		
Research papers	☐		☐		☐						☐						☐		
Medical books	☐		☐		☐						☐						☐		
The National Formulary	☐		☐		☐						☐						☐		
Clinical Guidelines	☐		☐		☐						☐						☐		
Pharmacopoeias (U.S., British)	☐		☐		☐						☐						☐		
